# Directed graph neural networks with partial directed coherence for seizure prediction and epileptogenic network characterization

**DOI:** 10.1016/j.isci.2026.117068

**Published:** 2026-08-10

**Authors:** Chenshuo Zhao, Hui Huang, Chao Fang, Hong Zhang, Suyue Zheng

**Affiliations:** 1 Department of Neurosurgery, The First Affiliated Hospital of Nanchang University, Nanchang, China; 2 Department of Neurosurgery, The Second Affiliated Hospital of Nanchang University, Nanchang, China; 3 Huankui College, Nanchang University, Nanchang, China

**Keywords:** seizure prediction, partial directed coherence, directed graph neural networks, epileptogenic network characterization, explainable artificial intelligence

## Abstract

While electroencephalography (EEG) analyses using undirected connectivity are well-established for seizure prediction and epileptogenic zone (EZ) network characterization, the complementary value of directed connectivity in graph neural networks (GNNs) remains unclear. We integrated partial directed coherence (PDC) graphs into a Digraph Inception Convolutional Network (DiGCN) to preserve directional edge information for seizure prediction, achieving accuracies of 96.11 ± 0.76%, 96.32 ± 0.70%, and 97.69 ± 0.67% on CHB-MIT, Siena, and the institutional cohort, respectively. In the institutional cohort, preictal modularity and small-world index were both significantly elevated (*p* < 0.001), reflecting increased modular segregation and topological reorganization. PDC in-degree was significantly elevated at EZ-concordant electrodes across five epilepsy subtypes (*p* < 0.05), whereas out-degree changes were not statistically significant. GNNExplainer identified compact, prediction-relevant subgraphs preserving these directed connectivity patterns. PDC-DiGCN provides an interpretable sensor-level framework for seizure prediction and EZ-concordant network characterization, though requiring larger prospective validation.

## Highlights

• A directed graph neural network predicts seizures from EEG recordings
• Preictal directed EEG networks show increased modularity and small-world index
• Preictal in-degree is elevated at electrodes concordant with the epileptogenic zone
• GNNExplainer identifies compact, prediction-relevant directed subgraphs

## Graphical abstract

**Figure:**
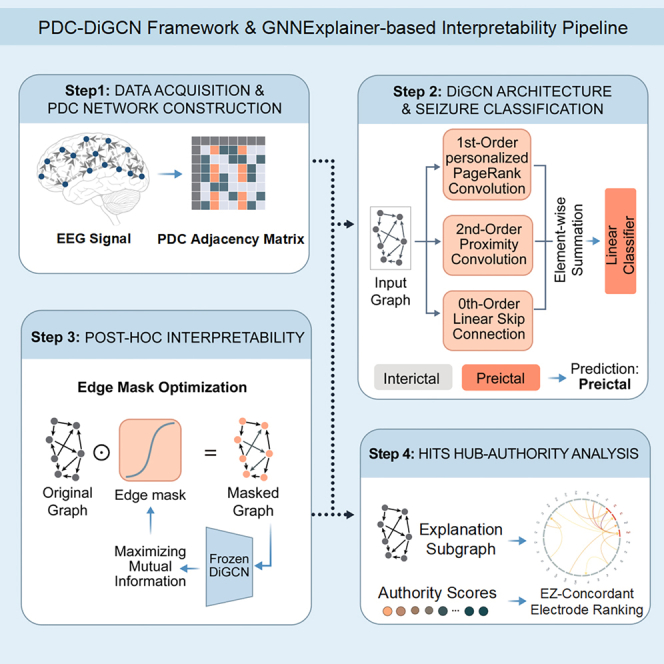

## Teaser

Health sciences; Medicine; Medical specialty; Internal medicine; Neurology

## Introduction

Drug-resistant focal epilepsy affects approximately one-third of the 65 million individuals with epilepsy worldwide,^1^^,^^2^^,^^3^ yet fewer than 25% of surgical candidates complete presurgical evaluation.^4^ Resective surgery achieves seizure freedom in 40–70% of appropriately selected patients,^4^^,^^5^ making accurate delineation of the epileptogenic zone (EZ) central to achieving favorable surgical outcomes. Scalp electroencephalography (EEG) remains the most widely used non-invasive modality in this presurgical pathway, offering high temporal resolution and clinical accessibility while reducing the procedural risks associated with intracranial recording. However, volume conduction and limited spatial resolution remain persistent challenges for electrode-level EZ characterization. These limitations highlight the need for a framework that can reduce spurious coupling and extract directionally informative network signatures from scalp EEG recordings.

Focal epilepsy is increasingly recognized as a disorder of large-scale directed networks, in which ictal discharges propagate hierarchically from the EZ through structurally constrained pathways to downstream propagation zones.^6^^,^^7^^,^^8^^,^^9^ Resection of the seizure onset zone (SOZ) rather than propagation regions predicts favorable surgical outcome.^10^ This clinical understanding has driven network-based approaches to characterize EZ-related epileptogenic network organization. Non-invasive network mapping has indicated that source-level functional connectivity can identify highly connected hubs within epileptogenic networks relevant to EZ characterization, with resection of these hubs associated with postoperative seizure freedom.^11^ The broader evidence base for network-based EZ characterization has primarily been built on undirected connectivity analyses and hub-strength measures.^9^^,^^12^^,^^13^ Neurophysiological evidence suggests that directed causal connectivity can reveal directional information flow within epileptogenic networks, and that such directionality is relevant to epileptogenic network characterization and surgical outcome.^10^ Ictogenic tissue also exhibits different directional roles across dynamic states, behaving as an information source characterized by high out-degree during ictal propagation.^14^ During seizure-free periods, the EZ receives sustained convergent input from surrounding cortex, producing elevated in-degree specific to ictogenic tissue.^15^^,^^16^ Whether directed causal connectivity provides information complementary to undirected approaches in EZ characterization remains to be systematically investigated.

Directed causal influences in multichannel EEG are commonly quantified using multivariate autoregressive (MVAR) modeling. In this framework, Granger causality is used to decompose inter-channel interactions into directed and frequency-resolved components.^17^^,^^18^ Among MVAR-derived estimators, partial directed coherence (PDC) and DTF are widely applied,^19^^,^^20^ but their normalization schemes emphasize different directional aspects of EZ-related network organization. Owing to row-wise normalization, DTF is more sensitive to outgoing network drive, whereas column-wise normalization makes PDC more sensitive to convergent inward connectivity and network sink behavior during non-ictal periods.^21^^,^^22^^,^^23^ Inward PDC measures have demonstrated greater EZ-related discriminative value than outward measures across cohorts.^15^^,^^16^^,^^21^^,^^23^ Grounded in Granger causality, PDC is considered relatively insensitive to volume conduction and robust to noise.^18^^,^^23^^,^^24^^,^^25^^,^^26^ This property is relevant to scalp EEG analysis, where spatial smoothing and volume conduction artifacts affect directed connectivity estimates. Supporting this advantage, prior scalp EEG studies have reported PDC-derived directed connectivity patterns associated with surgical resection sites and postsurgical outcome.^27^^,^^28^^,^^29^

Graph neural networks (GNNs) offer a natural computational framework for modeling structured inter-regional interactions and have achieved competitive benchmark performance in seizure prediction.^30^^,^^31^^,^^32^^,^^33^^,^^34^^,^^35^ Applying graph learning to PDC-derived directed graphs raises two methodological challenges. The first challenge concerns model architecture. Standard spectral graph convolutional network (GCN) formulations are defined through the eigendecomposition of a symmetric normalized Laplacian.^36^^,^^37^ Applying these formulations to directed graphs typically requires symmetrization,^38^ which discards the directional asymmetry that constitutes the principal information content of PDC-derived graphs. The Digraph Inception Convolutional Network (DiGCN) addresses this limitation by deriving a directed graph Laplacian from the personalized PageRank stationary distribution, thereby enabling a well-defined spectral formulation for directed graphs.^38^^,^^39^ The Inception-style parallel module is designed to capture multi-scale connectivity patterns and reduce the risk of over-smoothing.^38^ Interpretability presents a second methodological challenge. In the present seizure-prediction and EZ-characterization task, model decisions should be auditable and interpretable in relation to the underlying neurophysiology.^40^^,^^41^ Gradient-based saliency methods have been reported to produce attributions that are statistically indistinguishable from random baselines^42^ and may be affected by gradient saturation when applied to discrete adjacency matrices.^43^^,^^44^^,^^45^ Standard surrogate methods such as LIME and SHAP do not explicitly account for graph structural information.^46^^,^^47^ GNNExplainer addresses these limitations by formulating explanation as a mutual information maximization problem.^45^ By identifying compact, task-relevant subgraphs that retain prediction-relevant information, it enables structured assessment of whether model decisions align with the PDC in-degree pattern at EZ-concordant electrodes.

**Figure 1:**
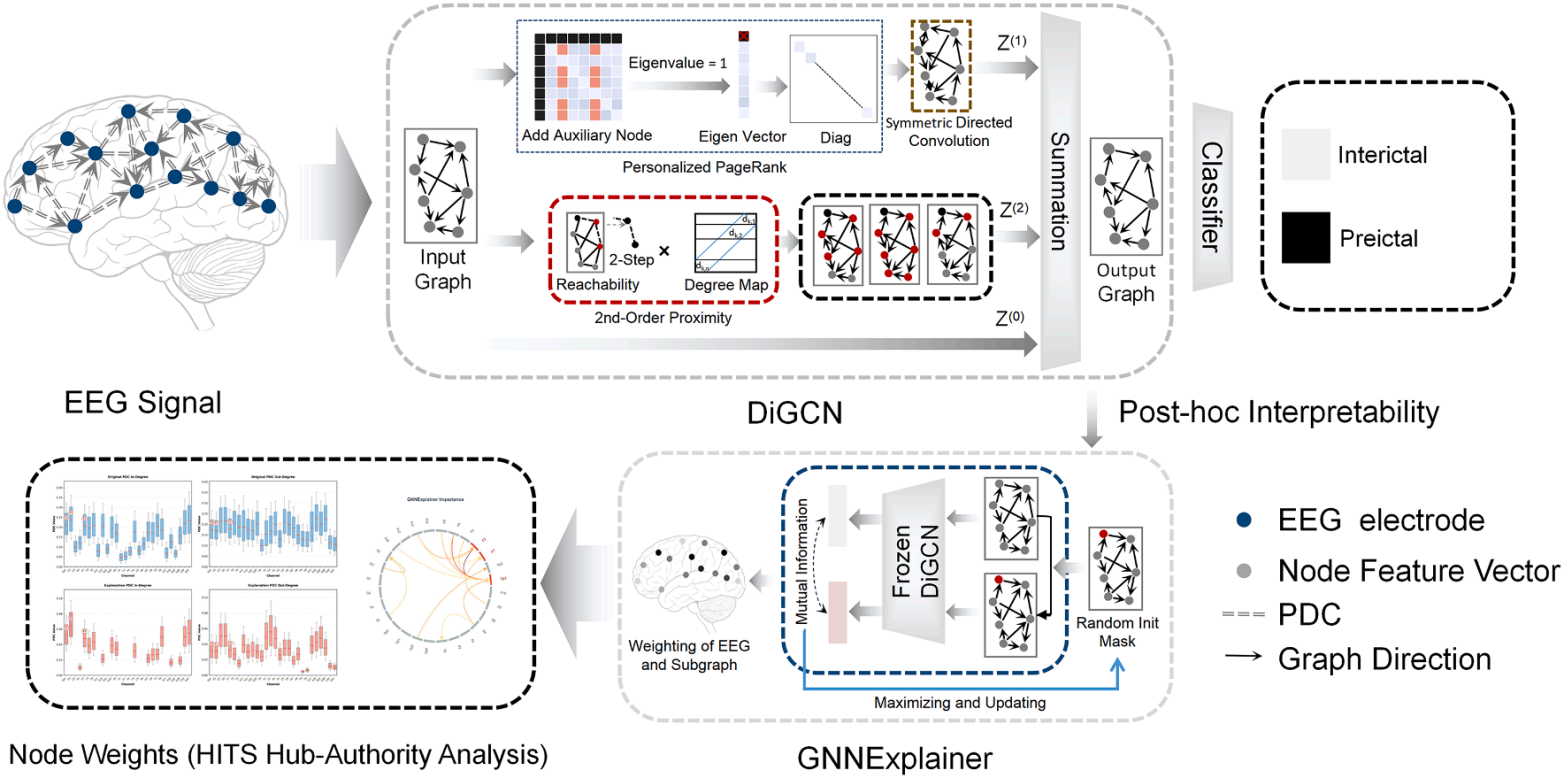
Schematic overview of the proposed PDC-DiGCN framework for seizure prediction and epileptogenic zone characterization Scalp EEG signals are processed into weighted directed graphs via partial directed coherence (PDC) and fed into the digraph inception convolutional network (DiGCN), which comprises three parallel streams fused within inception blocks. The first stream performs personalized PageRank-based directed graph convolution: an auxiliary teleport node is appended to the row-normalized transition matrix, the left eigenvector corresponding to eigenvalue 1 (the Perron vector) is computed, the auxiliary-node component is discarded, and the remaining components are l1-normalized to give the approximate stationary distribution; the corresponding diagonal matrix is used to symmetrize the forward propagation term and its transpose-based counterpart, whose average forms the symmetric normalized digraph convolution operator. The second stream computes second-order proximity convolution by intersecting the meeting-path and diffusion-path matrices element-wise (retaining the average only where both are strictly positive), followed by symmetric degree normalization. The third stream is a linear skip connection without graph smoothing. The three streams are fused by element-wise summation within each Inception block; the fused output is aggregated into a graph-level representation and passed to a linear classifier for interictal versus preictal classification. For post-hoc interpretability, GNNExplainer is applied to the trained and frozen DiGCN by optimizing a randomly initialized edge mask to maximize the mutual information between the model prediction and the masked subgraph; the optimized mask is element-wise multiplied with the original PDC weights to yield edge-level importance, and the Hyperlink-Induced Topic Search (HITS) algorithm subsequently computes hub and authority scores characterizing EEG electrodes as directed causal drivers (hubs) or convergent information sinks (authorities).

The present study evaluates PDC-derived directed graphs within a DiGCN framework using the CHB-MIT and Siena public databases and an institutional cohort of 68 patients (Figure 1), all of whom achieved Engel Class Ia outcome after at least 12 months of postoperative follow-up (Table 1). We assessed seizure-prediction performance against representative baselines and explored the associated preictal topological reorganization. In the institutional cohort, we further examined elevated preictal PDC in-degree at EZ-concordant electrodes and the preservation of this directed convergence pattern within GNNExplainer-derived subgraphs. Throughout the study, model outputs are interpreted as sensor-level EEG evidence consistent with an inward-directed connectivity pattern at EZ-concordant electrodes, rather than as definitive cortical EZ localization. Prospective validation incorporating source-localization procedures and postsurgical outcome data will be necessary to establish the clinical utility.

**Table 1:** Demographic and clinical characteristics of the study cohort

Variable		Total	Bilateral FLE	Right FLE	Right TLE	Left FLE	Left TLE	*p* value
Structural lesion subtype, n (%)	MTLE-HS	28	0	0	14	0	14	–
	FCD Type II	40	14	13	0	13	0	–
Age (years)	mean ± SD	36.1 ± 10.0	36.4 ± 10.7	33.8 ± 10.3	39.0 ± 10.7	32.6 ± 8.7	38.1 ± 9.5	0.413
	median [IQR]	36 [28–44]	36 [29–44]	33 [27–41]	38 [31–47]	33 [26–39]	38 [32–44]	0.470
	range	18–58	19–55	18–52	22–58	19–46	22–55	–
Sex, n (%)	male	42	9	8	9	7	9	0.977
	female	26	5	5	5	6	5	–
Epilepsy duration (years)	mean ± SD	9.1 ± 3.7	8.5 ± 3.9	8.1 ± 3.3	10.5 ± 4.1	7.7 ± 3.0	10.6 ± 3.7	0.115
	median [IQR]	9.1 [6.1–11.8]	7.9 [5.0–11.4]	7.6 [5.0–11.0]	10.5 [7.1–13.8]	7.2 [5.0–10.0]	10.5 [7.9–13.6]	0.164
	range	3.7–18.3	3.7–15.2	3.8–14.2	4.8–18.3	3.5–13.1	5.3–17.6	–
Pre-surgical ASM regimen, n (%)	dual therapy (2 ASMs)	52	9	10	12	9	12	0.581
	triple therapy (3 ASMs)	16	5	3	2	4	2	–
Primary seizure type (ILAE 2017)	focal aware (FA)	9	2	2	2	2	1	0.854
	focal impaired awareness (FIA)	57	12	10	12	10	13	–
	focal to bilateral tonic-clonic (FBTC)	2	0	1	0	1	0	–
Recorded seizure events per patient	range	5–9	5–8	5–8	5–9	5–9	5–9	–

## Results

### Seizure prediction performance across datasets

**Figure 2:**
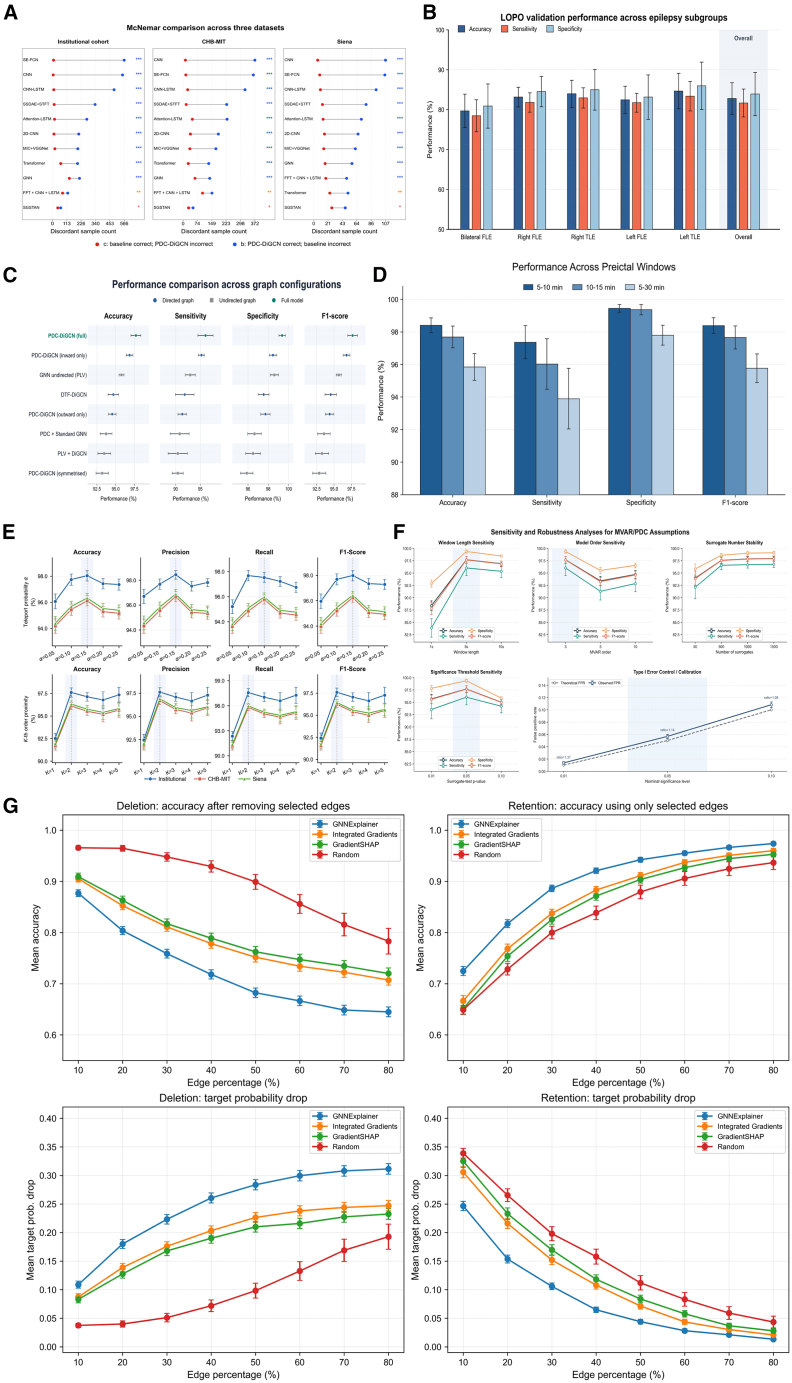
Validation and robustness analyses of the PDC-DiGCN framework (A) McNemar’s test with Holm–Bonferroni correction comparing PDC-DiGCN against 11 baseline models across three datasets. For each dataset, the comparison was performed on the held-out test set: institutional cohort, *n* = 8,160 epochs; CHB-MIT, *n* = 4,752 epochs; Siena, *n* = 1,128 epochs. For each pairwise comparison, b (n_10_) and c (n_01_) denote the numbers of discordant test samples (b: PDC-DiGCN correct, baseline incorrect; c: baseline correct, PDC-DiGCN incorrect), and the χ^2^ statistic was computed from these discordant pairs; *p* values were adjusted using the Holm–Bonferroni correction across the 11 pairwise comparisons within each dataset. ∗∗∗*p* < 0.001, ∗∗*p* < 0.01, and ∗*p* < 0.05. (B) Leave-one-patient-out (LOPO) cross-validation performance across five epilepsy subgroups. For each subgroup, each patient was in turn held out for testing while the remaining patients within the same subtype were used for training: bilateral FLE, *n* = 14; right FLE, *n* = 13; right TLE, *n* = 14; left FLE, *n* = 13; left TLE, *n* = 14; and overall, *n* = 68. Bars represent mean accuracy, sensitivity, and specificity for each subgroup and for the overall cohort. Error bars indicate the standard deviation across patients within each subgroup. (C) Performance comparison across eight graph configurations on the institutional cohort, evaluated to decouple the contributions of the graph construction metric (PDC, DTF, or PLV) and the learning architecture (DiGCN or undirected GNN): full directed PDC-DiGCN, inward-only directed PDC-DiGCN, outward-only directed PDC-DiGCN, DTF-DiGCN, PDC with standard GNN, PLV with DiGCN, PLV-based undirected GNN, and symmetrized PDC-DiGCN. Bars represent mean accuracy, sensitivity, specificity, and F1-score across *n* = 5 independent runs. Error bars indicate the standard deviation across the five runs. (D) Classification performance of the PDC-DiGCN framework across three preictal window lengths (5–10 min, 10–15 min, and 5–30 min) on the institutional cohort. Bars represent mean accuracy, sensitivity, specificity, and F1-score across *n* = 5 independent runs. Error bars indicate the standard deviation across the five runs. (E) Hyperparameter sensitivity of the personalized PageRank teleportation probability α (α ∈ {0.05, 0.10, 0.15, 0.20, 0.25}) and the K-th order proximity aggregation parameter K (K ∈ {1, 2, 3, 4, 5}) across the institutional, CHB-MIT, and Siena cohorts. Shaded vertical bars indicate the optimal parameter value for each metric (α = 0.15, K = 2). Data points represent the mean across *n* = 5 independent runs per condition; error bars indicate the standard deviation across the five runs. (F) Sensitivity and robustness analyses for MVAR-PDC graph-construction assumptions on the institutional cohort, examining epoch lengths of 1 s, 5 s, and 10 s; MVAR model orders of *p* = 3, 5, and 10; surrogate counts of *N* = 50, 500, 1000, and 1500; and surrogate-test thresholds of 0.01, 0.05, and 0.10, together with Type I error calibration using null data to assess the alignment between observed and nominal false-positive rates across nominal significance levels of 0.01, 0.05, and 0.10. Data points represent the mean across *n* = 5 independent runs per condition; error bars indicate the standard deviation across the five runs. (G) Edge ablation analysis compares four edge importance scoring methods (GNNExplainer, Integrated Gradients, GradientSHAP, and a random baseline) across edge percentages from 10% to 80%, evaluated on the held-out test set of the institutional cohort. Two complementary evaluation modes are reported: Deletion (left column) removes the top-ranked edges identified by each method, while Retention (right column) keeps only the top-ranked edges and removes the rest. Two metrics are measured under each mode: mean classification accuracy (top row) and mean target probability drop (bottom row). Data points represent the mean across *n* = 5 independent runs; error bars indicate the standard deviation across the five runs.

PDC-DiGCN achieved the highest accuracy among all eleven representative baseline architectures on the institutional cohort, reaching 97.69 ± 0.67% (Table 2). To further validate this advantage across independent datasets, McNemar’s test with Holm–Bonferroni correction confirmed statistically significant superiority across all three datasets—the institutional cohort, CHB-MIT, and Siena (Figure 2A). Model performance remained consistent across ten independent random seed configurations (Table S1), confirming that the reported results are robust to random initialization. On the CHB-MIT and Siena benchmark databases, the framework achieved mean accuracies of 96.11 ± 0.76% and 96.32 ± 0.70%, respectively (Tables S2 and S3), demonstrating consistent generalization across heterogeneous pediatric and adult acquisition environments. At the event level, the framework achieved a mean false alarm rate of 0.16 FP/h (false positives per hour) and a mean prediction latency of 14.07 min within the institutional cohort of 68 patients. This false alarm rate is consistent with the range reported by previously published scalp EEG-based seizure prediction studies (0.11–0.39 FP/h).^48^^,^^49^^,^^50^^,^^51^ The mean prediction latency of 14.07 min provides a window during which patients and caregivers may undertake protective responses prior to seizure onset. To assess patient-independent generalization under a more stringent evaluation scheme, leave-one-patient-out (LOPO) cross-validation was performed within each epilepsy subtype of the institutional cohort. PDC-DiGCN achieved an overall mean accuracy of 82.79 ± 3.97% across all 68 patients (Figure 2B), reflecting the inherent challenge of inter-subject EEG variability under patient-independent evaluation paradigms. This 14.90 percentage-point decline relative to the within-subject accuracy is of similar magnitude to the decline reported for a comparable graph-based seizure-prediction architecture evaluated under leave-one-subject-out protocols (14.1–16.2 percentage points).^52^ Reductions from standard cross-validation to LOPO evaluation can vary considerably depending on model complexity, dataset heterogeneity, and the use of patient-specific calibration.^53^^,^^54^

**Table 2:** Predictive performance of the proposed framework and baseline models

Model	Accuracy (%)	Sensitivity (%)	Specificity (%)	F1-score (%)	AUC (%)
SE-FCN	90.32 ± 1.11	84.18 ± 2.79	96.51 ± 0.86	89.70 ± 1.38	98.04 ± 0.23
CNN-LSTM	91.07 ± 0.59	84.63 ± 1.87	97.57 ± 0.87	90.49 ± 0.74	98.33 ± 0.15
GNN	95.77 ± 0.34	92.96 ± 1.05	98.61 ± 0.42	95.67 ± 0.38	99.49 ± 0.01
Attention-LSTM	93.85 ± 0.90	90.53 ± 2.86	97.21 ± 1.72	93.65 ± 1.06	99.00 ± 0.08
SSDAE + STFT	93.28 ± 1.74	88.99 ± 5.10	97.60 ± 1.86	92.94 ± 2.06	99.01 ± 0.17
MIC + VGGNet	94.71 ± 0.27	91.52 ± 0.93	97.94 ± 0.99	94.56 ± 0.27	99.24 ± 0.12
FFT + 1D-CNN	96.47 ± 0.31	95.50 ± 1.35	97.45 ± 0.98	96.45 ± 0.34	99.52 ± 0.08
2D-CNN	94.58 ± 0.75	90.62 ± 2.06	98.57 ± 0.66	94.37 ± 0.84	99.23 ± 0.15
CNN	90.12 ± 1.09	87.07 ± 2.81	93.17 ± 0.94	89.81 ± 1.24	97.55 ± 0.29
Transformer	95.23 ± 0.79	92.31 ± 2.27	98.15 ± 0.81	95.09 ± 0.87	99.08 ± 0.21
SGSTAN	96.14 ± 0.65	93.88 ± 1.94	98.40 ± 0.63	96.05 ± 0.72	99.46 ± 0.16
PDC-DiGCN	97.69 ± 0.67	96.03 ± 1.55	99.37 ± 0.32	97.66 ± 0.71	99.87 ± 0.03

Mean ± std (%) over 5 independent runs.

### Component and configuration ablation analyses

To evaluate the contribution of each architectural and graph construction component, performance was compared across eight graph configuration conditions and three architectural ablation conditions on the institutional cohort.

Configuration comparison evaluated eight conditions to decouple the contributions of the graph construction metric and the learning architecture: the full directed PDC-DiGCN, inward-only directed PDC-DiGCN, outward-only directed PDC-DiGCN, DTF-DiGCN, PDC with standard GNN, PLV with DiGCN, PLV-based undirected GNN, and symmetrized PDC-DiGCN (Figure 2C). The full PDC-DiGCN achieved the highest accuracy across all configurations, and the inward-only configuration yielded higher accuracy than the outward-only configuration, a pattern consistent with the role of PDC in-degree as a preictal directed-connectivity feature at epileptogenic nodes, though this difference should be interpreted with caution. Substituting either the PDC metric or the DiGCN architecture with its undirected counterpart resulted in performance reductions relative to the full model, suggesting that each component makes a separable contribution to classification performance under the conditions examined.

Component ablation analysis systematically removed one architectural module at a time, with all other settings held fixed across five independent runs on the institutional cohort (Table 3). Removing the symmetric bidirectional formulation of the digraph convolution operator (w/o symmetric) decreased accuracy from 97.73 ± 0.44% to 96.33 ± 0.43%, supporting the contribution of jointly encoding the forward transition term and its transpose-based counterpart, which together constitute the symmetric normalized propagation component of the directed graph Laplacian used in DiGCN. Removing the personalized PageRank-derived structural weighting (w/o PageRank) resulted in a larger reduction to 94.17 ± 0.46%, supporting the contribution of the approximate stationary distribution derived from the personalized PageRank auxiliary-node scheme to structurally weighted propagation and discriminative representation learning. Removing the higher-order proximity aggregation (w/o high-order) yielded the largest performance reduction, reducing accuracy to 92.74 ± 0.55%, supporting the contribution of the second-order proximity mechanism as implemented in the present model, which captures structural similarity between nodes sharing second-order directed neighborhood structure, operationalized through the simultaneous existence of meeting and diffusion paths via shared intermediate neighbors, within a single Inception block without requiring additional stacked convolutional layers. Collectively, these results indicate that each component makes a separable contribution to classification performance under the conditions evaluated.

**Table 3:** Component ablation study: contribution of each architectural module

Configuration	Accuracy (%)	Precision (%)	Recall (%)	F1-score (%)
Full model	97.73 ± 0.44	97.72 ± 0.43	97.70 ± 0.44	97.71 ± 0.41
w/o symmetric	96.33 ± 0.43	96.30 ± 0.44	96.36 ± 0.42	96.33 ± 0.42
w/o PageRank	94.17 ± 0.46	94.19 ± 0.47	94.17 ± 0.47	94.18 ± 0.46
w/o high-order	92.74 ± 0.55	92.74 ± 0.55	92.70 ± 0.54	92.72 ± 0.57

Mean ± std (%) over 5 independent runs.

### Hyperparameter and methodological sensitivity analyses

To characterize the sensitivity of the framework to key hyperparameter and methodological choices, systematic analyses were conducted across three domains: preictal window length, DiGCN architectural parameters, and MVAR-PDC estimation assumptions.

The effect of preictal window definition on model performance was evaluated under 5–10 min, 10–15 min, and 5–30 min conditions (Figure 2D). Accuracy remained high across all three settings: 98.41 ± 0.45%, 97.69 ± 0.67%, and 95.85 ± 0.82%, respectively. The 10–15 min window was selected as the primary configuration, given its comparable performance and more clinically actionable warning horizon relative to the 5–10 min condition. The 5–30 min window yielded a slightly reduced accuracy, suggesting that the framework maintained robust predictive performance across a broader preictal interval.

Hyperparameter sensitivity was assessed for the proximity order K and the personalized PageRank teleport probability α, with all other settings fixed and five independent runs per configuration (Figure 2E). K was evaluated over {1, 2, 3, 4, 5} across the institutional cohort, CHB-MIT, and Siena, and K = 2 was selected as the default setting based on the highest observed overall performance. The teleport probability α in personalized PageRank governs the trade-off between local neighborhood aggregation and global graph connectivity during directed message passing. α was evaluated over {0.05, 0.10, 0.15, 0.20, 0.25}, and α = 0.15 was selected as the default setting. These analyses were used to determine the default hyperparameter configuration for subsequent experiments, while optimal values should be adjusted for the dataset under investigation.

To evaluate robustness to key MVAR-PDC graph-construction choices, sensitivity analyses were performed for epoch length, model order, surrogate count, and surrogate-test threshold, together with Type I error calibration to quantify false-positive control (Figure 2F). Across epoch lengths of 1 s, 5 s, and 10 s, the 5 s epoch yielded the highest performance (Accuracy: 97.69 ± 0.67%), consistent with a balance between MVAR estimation reliability and the local stationarity requirement. The AIC-selected model order of *p* = 3 performed best, supporting the use of data-driven model-order selection in the present framework. Surrogate count stability was evaluated across *N* = 50, 500, 1000, and 1500; performance was lower at *N* = 50 and stabilized from *N* = 500 onward, with limited additional improvement at higher counts, supporting the use of *N* = 1000 as a practical default for surrogate-based null estimation. The surrogate-test threshold of 0.05 yielded the highest overall performance, with lower performance observed at both 0.01 and 0.10. Type I error calibration using null data showed that the observed false-positive rate remained broadly aligned with nominal levels, particularly at the adopted 0.05 threshold (ratio = 1.14), with the largest inflation observed at the most stringent threshold. Collectively, these analyses support the robustness of the proposed framework across the tested MVAR-PDC parameter ranges and provide empirical justification for the adopted MVAR-PDC graph-construction configuration.

### GNNExplainer edge ablation analysis

**Figure 3:**
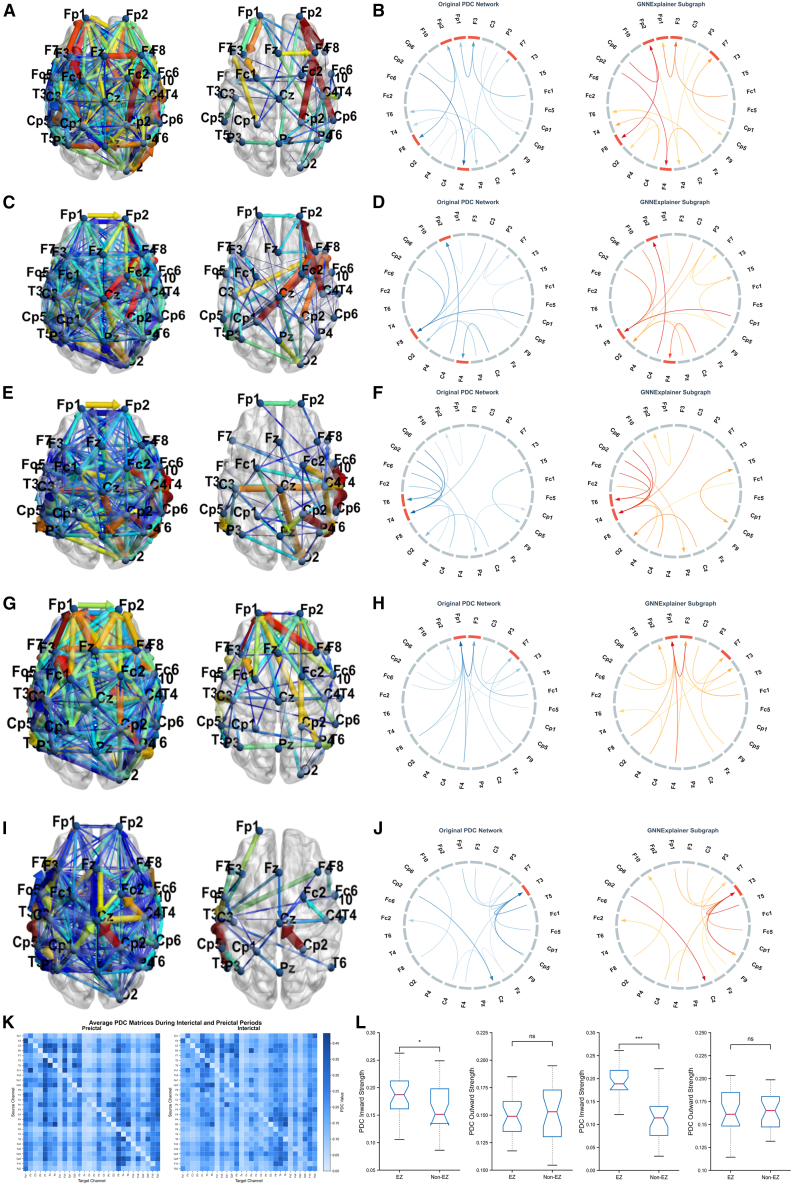
Preictal PDC-based directed brain connectivity patterns across five epilepsy subtypes (A and B) Bilateral frontal epilepsy: (A) BrainNet Viewer visualizations of the original PDC network (left) and GNNExplainer explanation subgraph (right); (B) chord diagrams showing the top 15 PDC-weighted directed edges in the original PDC network (left) and explanation subgraph (right), with red arc segments indicating EZ-concordant electrodes and darker line color indicating higher edge weight. (C and D) Right frontal epilepsy, displayed in the same format as in (A–B). (E and F) Right temporal epilepsy, displayed in the same format as in (A–B). (G and H) Left frontal epilepsy, displayed in the same format as in (A–B). (I and J) Left temporal epilepsy, displayed in the same format as in (A–B). (K) PDC connectivity matrices during the preictal (left) and interictal (right) periods from a single representative patient, illustrating the spatial reorganization of directed connectivity between the two states. (L) PDC inward strength and outward strength for EZ-concordant versus non-EZ-concordant electrodes during the interictal period (left two panels) and preictal period (right two panels), based on *n* = 8,160 epochs from the held-out test set, nested within 68 patients of the institutional cohort. Statistical comparisons were performed using linear mixed-effects models with patient identity included as a random intercept to account for within-patient dependence among repeated epoch-level observations. ∗*p* < 0.05 and ∗∗∗*p* < 0.001; ns, not significant.

**Figure 4:**
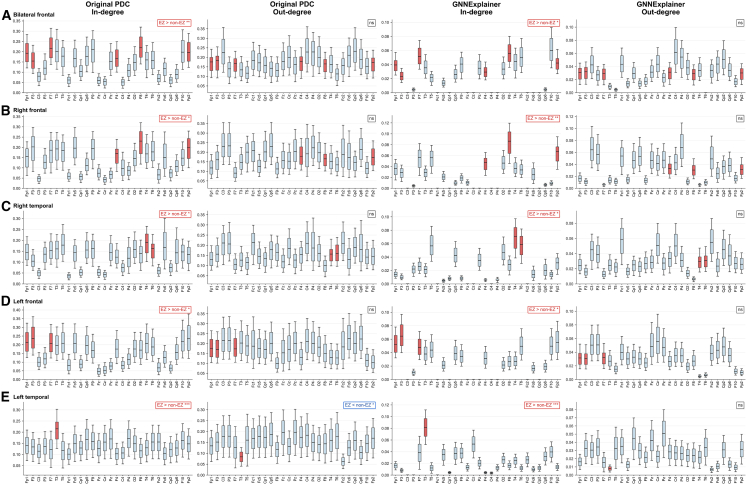
Electrode-level PDC in-degree and out-degree distributions across five epilepsy subtypes in the original PDC network and GNNExplainer explanation subgraph (A) Bilateral frontal epilepsy. (B) Right frontal epilepsy. (C) Right temporal epilepsy. (D) Left frontal epilepsy. (E) Left temporal epilepsy. For each electrode, PDC in-degree (the summed weight of incoming directed connections) and out-degree (the summed weight of outgoing directed connections) were computed separately for the original PDC network and the corresponding GNNExplainer-derived explanation subgraph, and compared between EZ-concordant and non-EZ-concordant electrodes within each subtype. Statistical comparisons were performed using linear mixed-effects models with patient identity included as a random intercept. Red boxplots indicate EZ-concordant electrodes; blue boxplots indicate non-EZ-concordant electrodes. ∗*p* < 0.05, ∗∗*p* < 0.01, and ∗∗∗*p* < 0.001; ns, not significant.

To assess the functional relevance of the directed edge structures identified by GNNExplainer, an edge ablation analysis was conducted comparing GNNExplainer against Integrated Gradients, GradientSHAP, and a random baseline across edge proportions of 10%–80% under both deletion and retention protocols (Figure 2G). Under the deletion protocol, GNNExplainer consistently produced a more pronounced decrease in classification accuracy and a greater target class probability drop relative to Integrated Gradients, GradientSHAP, and the random baseline across the full range of proportions examined. Under the retention protocol, GNNExplainer maintained superior predictive accuracy and a lower target class probability drop across the same range, demonstrating that the selected edge subset better preserved predictive performance. Considered alongside the preferential spatial convergence of explanation-identified edges onto EZ-concordant electrodes (Figures 3A–3J) and the consistent in-degree elevation at EZ-concordant nodes following GNNExplainer-based sparsification (Figures 4A–4E), these findings provide convergent evidence that the directed connectivity structure identified by GNNExplainer is functionally associated with predictive accuracy and directionally consistent with the in-degree elevation pattern observed at EZ-concordant electrodes.

### Multi-scale network topology analysis

Eight graph-theoretic metrics were computed for interictal and preictal epochs across both full PDC networks and GNNExplainer explanation subgraphs: network density, mean degree, modularity, reciprocity, degree distribution variance, clustering coefficient, characteristic path length, and small-world index. All eight metrics showed statistically significant interictal-to-preictal transitions in both network representations (linear mixed-effects model with patient identity as random intercept; all *p* < 0.001). Critically, the directional pattern of change was concordant across full PDC networks and explanation subgraphs, indicating that the connectivity features driving DiGCN classification are associated with the preictal reorganization observed at the network level.

**Figure 5:**
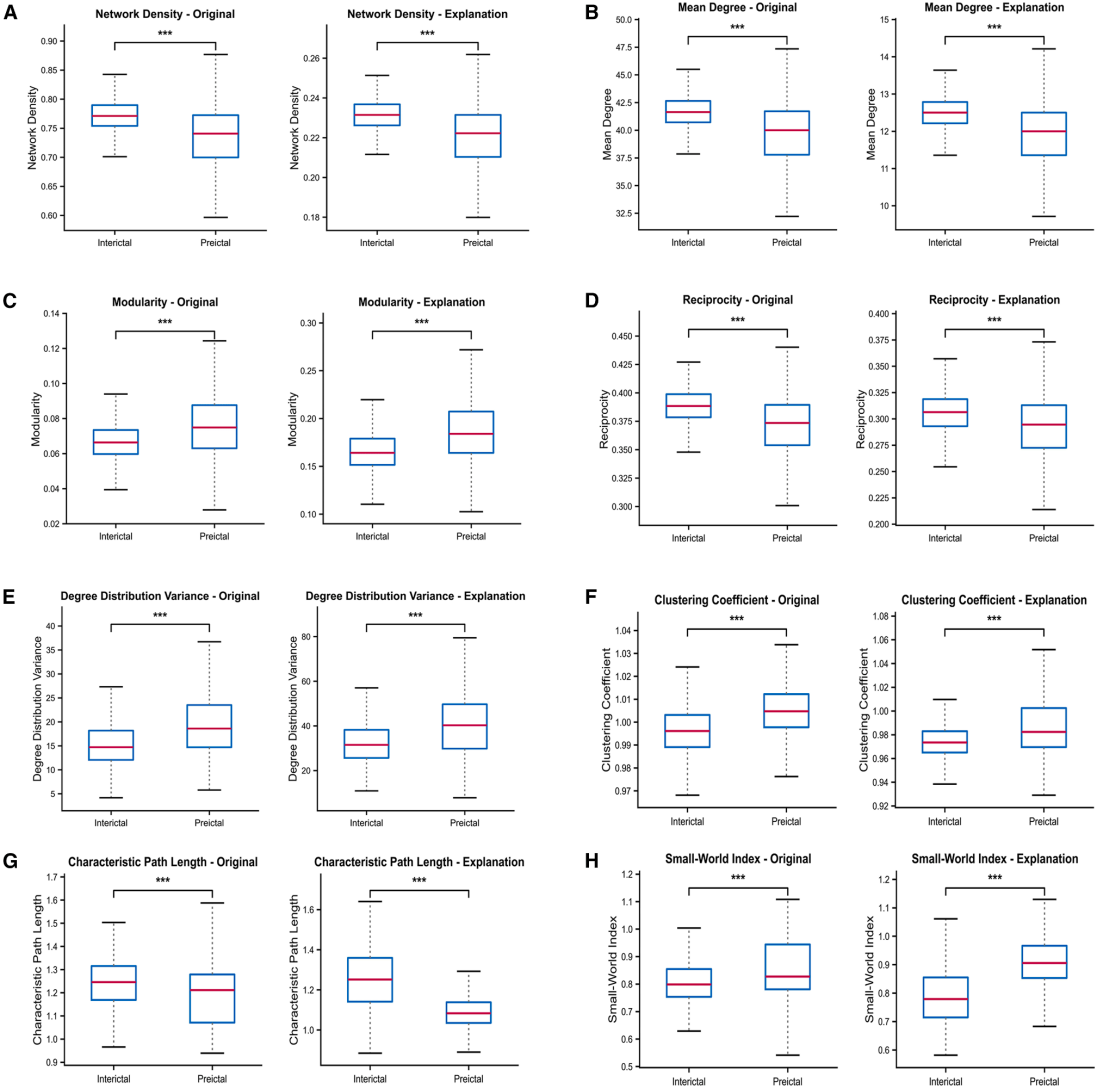
Network topology metrics compare interictal and preictal states across full PDC networks and GNNExplainer explanation subgraphs (A) Network density, reflecting the proportion of realized directed connections among all possible directed node pairs. (B) Mean degree, reflecting the average total connectivity of each node based on incoming and outgoing directed connections. (C) Network modularity, reflecting the extent to which the network is organized into densely interconnected sub-communities relative to a null model. (D) Network reciprocity, reflecting the extent to which directed connections between electrodes are reciprocated by edges in the opposite direction. (E) Degree distribution variance, reflecting the dispersion of nodal degree across electrodes and the associated heterogeneity in directed connectivity. (F) Clustering coefficient, reflecting the tendency of neighboring nodes to form locally interconnected structures. (G) Characteristic path length, reflecting the average shortest path length between node pairs. (H) Small-world index, reflecting the balance between local clustering and global network integration relative to matched random networks. For each panel, the left boxplots show the original PDC network, whereas the right boxplots show the corresponding GNNExplainer-derived explanation subgraph. Interictal and preictal states were compared within each network representation, based on the held-out test set of the institutional cohort (*n* = 8,160 epochs nested within 68 patients; 4,080 preictal and 4,080 interictal). Statistical comparisons were performed using linear mixed-effects models with patient identity included as a random intercept; ∗∗∗*p* < 0.001.

Network density and mean degree were significantly reduced in the preictal state relative to the interictal baseline (both *p* < 0.001; Figures 5A and 5B), whereas modularity was significantly increased (*p* < 0.001; Figure 5C). This pattern suggests a preictal network reconfiguration from diffuse network-wide coupling toward spatially constrained subnetworks. Preictal iEEG network analyses have similarly shown that SOZ contacts exhibit an increased proportion of within-group connections during the preictal transitional state, whereas surrounding non-SOZ contacts remain relatively stable.^55^ A computational modeling study by Woldman et al. further indicates that focal seizure emergence depends on the interaction between network architecture and spatial heterogeneity in regional excitability, rather than on uniformly distributed ictogenic potential across the network.^56^ This pattern is also consistent with graph-theoretical evidence from focal epilepsy showing increased clustering, local efficiency, and modularity, supporting a more locally compartmentalized network organization in epileptic brains.^57^^,^^58^^,^^59^^,^^60^ Collectively, these findings support the view that the preictal state is associated with a more segregated and spatially heterogeneous directed network configuration.

Reciprocity was significantly reduced in the preictal state (*p* < 0.001; Figure 5D), whereas degree distribution variance was significantly increased (*p* < 0.001; Figure 5E). Lower reciprocity indicates fewer bidirectional edge pairs, suggesting a more asymmetric organization of information flow, while increased degree variance suggests that directed connectivity becomes concentrated within a restricted subset of nodes. This interpretation is supported by preictal iEEG evidence showing that degree centrality increases at SOZ contacts before seizure onset, relative to simultaneous activity at non-SOZ contacts and interictal activity at the same SOZ contacts, whereas non-SOZ regions show no comparable change.^55^ Evidence of asymmetric functional disconnection involving the SOZ further supports the relevance of directionality in epileptic network organization.^61^

Concomitant with the above directional changes, the clustering coefficient was significantly increased in the preictal state (*p* < 0.001; Figure 5F), whereas characteristic path length was significantly reduced (*p* < 0.001; Figure 5G), resulting in a significant increase in the small-world index (*p* < 0.001; Figure 5H). This topological profile suggests that the preictal directed network is characterized by increased local clustering and a more prominent small-world organization. Increased clustering is consistent with prior graph-theoretical studies of focal epilepsy showing enhanced local network organization and greater topological regularity in epileptic networks, particularly during preictal states.^57^^,^^62^ Similar preictal small-world reorganization has also been observed in scalp EEG networks, where beta-band networks showed higher clustering coefficient and shorter characteristic path length before seizure onset relative to the interictal state.^63^

Considered collectively, the eight topological metrics indicate increased modular compartmentalization, directional asymmetry, and topological heterogeneity in the preictal sensor-space directed network. The consistency of these changes across full PDC networks and GNNExplainer-derived subgraphs suggests that the explanatory subgraphs preserve the principal sensor-space directed connectivity patterns observed during the preictal state.

### Node-aggregated PDC connectivity reveals EZ-concordant electrodes as preferential directed information sinks across seizure onset subtypes

To interrogate the neurophysiological basis of DiGCN-based classification, per-node PDC in-degree and out-degree were computed as graph-theoretic indices of directed information flow across five clinically distinct epilepsy subtypes: bilateral frontal, right frontal, right temporal, left frontal, and left temporal lobe epilepsies. BrainNet Viewer visualization of the PDC edge networks showed that, across all five subtypes, directed connectivity preferentially converged onto EZ-concordant electrodes in both the original PDC network and the GNNExplainer-derived explanation subgraph (Figures 3A–3C, 3E, and 3G). Complementary chord-plot visualization further showed preferential termination of high-weight directed edges at EZ-concordant nodes (Figures 3B–3D, 3F, and 3H). The PDC heatmaps reveal that preictal directed inward connectivity becomes more spatially organized compared with the interictal state (Figure 3K). Statistical comparison of PDC inward and outward strength between EZ-concordant and non-EZ-concordant electrodes indicated that, in the interictal period, inward strength was significantly higher at EZ-concordant electrodes than at non-EZ-concordant electrodes (*p* < 0.05), while outward strength showed no significant difference; in the preictal period, this inward strength difference was statistically more pronounced (*p* < 0.001), while outward strength remained non-significant (Figure 3L). This pattern indicates that EZ-concordant electrodes exhibit inward-directed convergence already present during the interictal baseline, consistent with related intracranial findings^15^^,^^21^^,^^64^ and potentially representing a stable property of EZ-concordant tissue during the non-ictal period. This co-occurs with the broader preictal network reorganization described above (Figure 5), which may be more preferentially associated with the preictal state than the EZ in-degree elevation considered in isolation.

Quantitative analysis supported this direction-specific pattern across all five subtypes (Figures 4A–4E). In-degree was significantly elevated at EZ-concordant electrodes in both the original network and the explanation subgraph across all subtypes. Out-degree differences did not reach statistical significance in either analytical stage for bilateral frontal, right frontal, right temporal, and left frontal subtypes (*p* > 0.05). In the left temporal subtype, out-degree was statistically reduced at EZ-concordant electrodes in the original network (*p* < 0.05), but this difference was not significant in the explanation subgraph (*p* > 0.05). This pattern is supported by independent intracranial cohorts. PDC inward strength at ictogenic regions has been reported to exceed that of non-EZ regions, whereas outward strength does not differ significantly across region types.^15^^,^^23^^,^^55^ In addition, the electrode contact with the highest PDC in-degree has been shown to localize within the clinically identified EZ.^21^ The inward–outward dissociation has also been reported to remain stable over seven consecutive days of interictal recording, independent of antiseizure medication dosage.^64^ From a neurophysiological perspective, this pattern is consistent with the EZ acting as a net receiver of directed input during the preictal period.^15^^,^^16^^,^^65^

## Discussion

The present study shows that scalp EEG connectivity analysis is affected by spatial smoothing and volume conduction, while PDC is relatively less sensitive to these effects and may reduce their influence on directed connectivity estimation. By integrating PDC-derived directed graphs with DiGCN, the proposed framework supports seizure prediction while preserving direction-resolved connectivity information. The higher predictive performance relative to symmetric graph configurations suggests that preserving the asymmetry of PDC adjacency matrices provides additional direction-resolved connectivity information for classifying preictal and interictal EEG. The consistently high performance across the institutional cohort, CHB-MIT, and Siena datasets further supports the robustness of this graph construction strategy for seizure prediction.

In the institutional cohort, topological analysis showed systematic preictal reorganization of sensor-level directed networks, including reduced network density and mean degree, increased modularity, reduced reciprocity, increased degree distribution variance, increased clustering coefficient, shortened characteristic path length, and increased small-world index. These changes indicate more pronounced modular segregation, directional asymmetry, and topological heterogeneity in the preictal network. Within this reorganized network context, EZ-concordant electrodes showed significantly elevated PDC in-degree during the preictal period, whereas out-degree changes did not reach statistical significance, indicating predominant inward convergence at EZ-concordant electrodes. GNNExplainer-derived subgraphs retained the topological transition pattern and the in-degree profile after sparsification, and edge ablation further showed that the retained directed edges were functionally associated with predictive performance. Together, these findings suggest that the edges identified by GNNExplainer contributed to model prediction and were associated with EZ-concordant directed network features, while remaining exploratory rather than causal evidence.

By combining PDC-derived directed graphs, DiGCN, and GNNExplainer, the framework retains the practical advantages of non-invasive and clinically accessible scalp EEG while linking seizure prediction with interpretable sensor-level network characterization. It integrates seizure-prediction output, preictal topological reorganization, and EZ-concordant directed connectivity features, allowing model interpretation to extend from classification accuracy to network topology and direction-resolved connectivity.

## Limitations of study

The present study has several limitations. First, the PDC-DiGCN outputs should be interpreted as exploratory EEG-level characterizations consistent with clinically defined EZ-concordant regions rather than as independent cortical-level EZ localization conclusions. Prospective validation in larger, multicenter cohorts incorporating ground-truth resection data and postsurgical outcome assessment is required before clinical utility can be established. Second, the present framework adopts a patient-specific modeling approach, in which training and test data are drawn from the same patient; such approaches do not directly assess generalization to unseen patients, and performance declines under LOPO cross-validation. Third, MVAR modeling assumes local stationarity of the EEG signal, and future work employing time-varying MVAR models may better capture the dynamic evolution of directed connectivity in the preictal period. Fourth, the present framework operates in sensor space, and concurrent intracranial EEG recordings were not obtained for the institutional cohort. Furthermore, node-level causal graphs were constructed directly at the scalp sensor level without prior application of source localization procedures. Scalp EEG is subject to an inherent physical limitation in that spatial smoothing and volume conduction artifacts adversely affect directed connectivity estimates extracted from PDC; although PDC’s Granger-causality-based formulation is considered to confer robustness to volume conduction, this robustness is partial rather than absolute. Direct iEEG validation of sensor-space PDC in-degree and the integration of source localization techniques prior to directed graph construction remain important priorities for future work.

## Resource availability

### Lead contact

Requests for further information and resources should be directed to and will be fulfilled by the lead contact, Suyue Zheng (ndyfy04043@ncu.edu.cn).

### Materials availability

This study did not generate new unique reagents.

### Data and code availability

• This paper analyzes existing, publicly available datasets. The CHB-MIT Scalp EEG Database is available at PhysioNet (https://physionet.org/content/chbmit/1.0.0/), and the Siena Scalp EEG Database is available at PhysioNet (https://physionet.org/content/siena-scalp-eeg/1.0.0/).
• The institutional cohort EEG data reported in this paper cannot be publicly deposited because of patient privacy, institutional data-sharing policies, and ethics restrictions. De-identified data are available from the lead contact upon reasonable request, subject to institutional approval and applicable data-sharing regulations.
• The original code implementing the PDC-DiGCN framework is publicly available at GitHub (https://github.com/chenshuozhao/PDC-DiGCN-Seizure-Prediction).
• Any additional information required to reanalyze the data reported in this paper is available from the lead contact upon request.

## Acknowledgments

This work was supported by the 10.13039/501100004479Jiangxi Provincial Natural Science Foundation (20242BAB25470) and the Jiangxi Provincial Health Commission Science and Technology grant no. (202210372). S.Z. (202406820104) and H.H. (202308360151) were supported by the China Scholarship Council as exchange scholars.

## Author contributions

C.Z., S.Z., and H.H.; conceived and designed the study. C.Z.; developed the PDC-DiGCN framework, implemented the GNNExplainer post-hoc interpretability pipeline, and conducted all computational and statistical analyses. H.H. and C.F.; contributed to the clinical interpretation of the directed connectivity findings and validated the PDC in-degree signature against established neurophysiological principles of epileptogenic network organization. H.Z.; provided expertise in epilepsy neurosurgery, guided the characterization of epileptogenic zones across seizure onset subtypes, and assessed the clinical relevance of the findings for presurgical evaluation. C.Z.; performed EEG data preprocessing, PDC graph construction, model training, and multi-scale network topology analysis. C.Z. and S.Z.; wrote the original draft of the manuscript. All authors contributed to manuscript revision and read and approved the submitted version. S.Z.; supervised the project and acquired funding.

## Declaration of interests

The authors declare no competing interests.

## STAR★Methods

### Key resources table

**Table:** 

REAGENT or RESOURCE	SOURCE	IDENTIFIER
**Software and algorithms**		

EEGLAB	Delorme^66^	RRID:SCR_007292
BrainNet Viewer	Xia^67^	RRID:SCR_009446
PDC-DiGCN code	This paper	https://github.com/chenshuozhao/PDC-DiGCN-Seizure-Prediction

**Deposited data**		

CHB-MIT Scalp EEG Database	Guttag^68^	https://doi.org/10.13026/C2K01R
Siena Scalp EEG Database	Detti^69^	https://doi.org/10.13026/5d4a-j060
Institutional cohort EEG data	This paper	N/A

### Experimental model and study participant details

#### Human participants

The institutional cohort comprised 68 patients with drug-resistant focal epilepsy (42 male, 26 female; mean age 36.1 ± 10.0 years, range 18–58 years), all of whom were followed up for at least 12 months and achieved Engel Class Ia outcome at the last follow-up (range 12–48 months; mean 24.6 ± 8.7 months). Subtype assignment across five epilepsy subtypes was defined according to the International League Against Epilepsy (ILAE) 2017 operational classification and corroborated by post-surgical outcome. All patients were classified as structural etiology; 28 were diagnosed with mesial temporal lobe epilepsy with hippocampal sclerosis (MTLE-HS) and selected for the temporal lobe epilepsy (TLE) subtypes, verified by hippocampal volumetry and/or post-surgical histopathological examination; 40 were diagnosed with focal cortical dysplasia (FCD) Type II and selected for the frontal lobe epilepsy (FLE) subtypes, classified per ILAE 2022 criteria and verified by MRI and/or post-surgical histopathological examination. EZ localisation was established through multidisciplinary team evaluation, with final delineation determined by consensus at a dedicated epilepsy surgery conference. No statistically significant differences were observed across the five epilepsy subtypes in age, sex, epilepsy duration, pre-surgical ASM regimen, or primary seizure type distribution, indicating satisfactory baseline comparability across groups. Demographic and clinical characteristics are summarised in Table 1.

Sex-stratified analyses were not performed due to limited sample size within each subgroup, and ancestry, race, ethnicity, and socioeconomic status were not collected for this cohort; these factors limit the generalisability of the findings to broader and more diverse populations.

This study was conducted in accordance with the ethical principles outlined in the Declaration of Helsinki and was reviewed and approved by the Ethics Committee of the First Affiliated Hospital of Nanchang University (Approval No.: (2026)CDYFYYLK(04–023)). All participants provided written informed consent prior to enrollment.

#### CHB-MIT scalp EEG database

The CHB-MIT Scalp EEG Database is a publicly available dataset comprising scalp EEG recordings from 23 pediatric patients (5 male, 18 female; ages 1.5–22 years) with intractable seizures, collected at Children’s Hospital Boston. Recordings contain 198 seizures across 664 EDF files using a standard 10–20 electrode system. Detailed descriptions of the CHB-MIT database have been provided previously,^70^ and the dataset is publicly available through PhysioNet (https://doi.org/10.13026/C2K01R).

#### Siena Scalp EEG database

The Siena Scalp EEG Database is a publicly available dataset comprising scalp EEG recordings from 14 patients with epilepsy (8 male, 6 female; ages 20–71 years), collected at the Unit of Neurology and Neurophysiology of the University of Siena, Italy. Recordings contain seizures across approximately 128 h of monitoring, acquired at 512 Hz using a standard 10–20 electrode system. Detailed descriptions of the Siena Scalp EEG database have been provided by Detti et al.,^69^ and the dataset is publicly available through PhysioNet (https://doi.org/10.13026/5d4a-j060). The Ethical Committee of the University of Siena approved the data collection in accordance with the Declaration of Helsinki, and written informed consent was obtained from all participants.

### Method details

#### EEG data preprocessing

Offline EEG preprocessing was performed using the EEGLAB toolbox. Power-line noise was attenuated using a 50-Hz notch filter. The continuous EEG data were then subjected to zero-phase band-pass filtering (0.5–50 Hz). Subsequently, independent component analysis (ICA) was applied to identify and remove ocular and muscular artifacts.^71^^,^^72^^,^^73^^,^^74^^,^^75^^,^^76^ The upper cutoff of 50 Hz was selected given the documented susceptibility of high-frequency scalp EEG signals to myogenic contamination.^77^^,^^78^ Preictal epochs were defined as the 5-min segments spanning from 15 to 10 min prior to the annotated seizure onset, and interictal epochs were defined as segments recorded more than 2 h from any annotated seizure event; interictal segments were randomly sampled to achieve a 1:1 class ratio relative to preictal epochs. All EEG segments were extracted using a 5-s window. All epoch boundaries were determined at the seizure-event level prior to data partitioning, whereby all segments derived from a given seizure event were assigned exclusively to a single partition; seizure events were then allocated to training, validation, and test sets in a 6:2:2 ratio via stratified random sampling. As each independent experimental run employed a distinct random event-level partition, minor variation in reported performance metrics reflects sampling variability across partitions rather than instability of the proposed framework.

#### Partial directed coherence analysis

To characterise the directional information flow among EEG electrode contacts during the peri-ictal period and thereby delineate the epileptogenic network, we employed partial directed coherence (PDC) as the measure of effective connectivity. PDC is an effective connectivity measure rooted in the framework of Granger causality that quantifies the fractional causal influence transmitted from one neural source to another at each frequency, while discriminating direct from indirect interactions.^20^ Let *X*(*t*)∈R^*N*^ denote the column vector of EEG signals recorded simultaneously across all unipolar electrode channels at time *t*, where *N* is the number of channels retained after preprocessing. A *p*-th order MVAR model was fitted to the multichannel data:

(Equation 1) $$X\left(t\right)=\sum_{r=1}^{p}\;A\left(r\right)X\left(t-r\right)+E\left(t\right)$$

where A(*r*)∈R^*N*×*N*^ are the lag-*r* coefficient matrices, and *E*(*t*) is a zero-mean multivariate white-noise innovation vector with covariance matrix Σ. The model order *p* was determined by the Akaike information criterion (AIC). Coefficient matrices were estimated via the Yule–Walker equations.

Once the MVAR coefficients had been obtained, they were mapped to the frequency domain by evaluating the *z*-transform on the unit circle. The spectral coefficient matrix was defined as:

(Equation 2) $$\bar{A}\left(f\right)=I-\sum_{r=1}^{p}\;A\left(r\right)e^{-j2\pi fr}$$

where I∈R^*N*×*N*^ is the identity matrix and *f* denotes frequency. The PDC from source channel *j* to target channel *i* at frequency *f* was then defined as:

(Equation 3) $$\pi_{ij}\left(f\right)=\frac{\left|{\bar{A}}_{ij}\left(f\right)\right|}{\sqrt{\sum_{k=1}^{N}\;{\left|{\bar{A}}_{kj}\left(f\right)\right|}^{2}}}$$

where ${\bar{A}}_{ij}$ denotes the (*i*, *j*)-th element of $\bar{A}\left(f\right)$, and the summation in the denominator runs over all channels *k* = 1, 2, …,*N*. By construction, *π*_*ij*_(*f*)∈[0,1], and the column-wise normalisation enforces $\sum_{i=1}^{N}\;\pi_{ij}^{2}\left(f\right)=1$ for every source channel *j*. PDC therefore quantifies the proportional contribution of the outgoing causal influence from source channel *j* that is directed to target channel *i*, thereby isolating direct causal pathways while suppressing indirect signal propagation.

Spurious causal connections arising from noise or non-stationarities were eliminated through a nonparametric surrogate-data procedure.^79^ For each analysis window, the EEG signals were Fourier transformed, the spectral phases were independently randomised across all channels, and the resulting surrogate realisations were reconstructed via the inverse Fourier transform. PDC was recomputed for each surrogate, and this procedure was iterated 1,000 times to generate an empirical null distribution under the hypothesis of no directed interaction. A pointwise significance threshold was set at *p* = 0.05; any PDC value failing to exceed the corresponding 95th percentile of its null distribution was set to zero. This yielded a total of 756 directed channel pairs (28 × 27 channels). Across 1,000 resampled preictal epochs, the mean number of surviving edges after surrogate thresholding was 554.07 ± 41.22, corresponding to a mean network density of 0.733 ± 0.055. Across 1,000 resampled interictal epochs, the mean number of surviving edges was 581.30 ± 20.99, corresponding to a mean network density of 0.769 ± 0.028. Across all 2,000 resampled epochs, the mean number of surviving edges was 567.68 ± 35.42, corresponding to a mean network density of 0.751 ± 0.047.

Following significance thresholding, the broadband peak PDC value was extracted for each ordered channel pair across the analyzed frequency range. The resulting matrix was transposed to obtain the weighted directed association matrix W:

(Equation 4) $$w_{ij}=\max_{f\in F}\;\pi_{ji}\left(f\right)$$

Each element *w*_*ij*_ of W encodes the peak directed causal influence from channel *i* to channel *j* integrated across the full recording bandwidth. W constitutes the adjacency representation of a fully connected, directed, weighted graph in which each unipolar EEG electrode channel corresponds to a node and each non-zero element to a weighted directed edge. Because PDC column-wise normalisation amplifies the detectability of convergent information sinks, in-degree emerges as the topological metric best aligned with the computational properties of PDC for EZ characterization.

Because MVAR modeling requires local stationarity, all computations were performed within a 5-s sliding window advanced in steps of 0.125 s, yielding a time-resolved sequence of association matrices W(t) that continuously tracked peri-ictal network dynamics. For EZ localisation, graph measures (in-degree, out-degree, and betweenness centrality) were accumulated over the 5-s epoch immediately preceding the visually identified seizure onset and normalised to the range [0, 1].

#### DiGCN architecture

The weighted directed association matrix W∈R^*n*×*n*^ constructed from PDC analysis encodes continuous-valued causal influences among all *n* unipolar EEG electrode channels. Rather than binarising W via a fixed threshold—which would discard the graded strength of inter-channel causal interactions—we feed the full weighted matrix directly into a Digraph Inception Convolutional Network(DiGCN). This choice preserves the graded causal structure encoded in the PDC matrix and allows the model to exploit fine-grained differences in PDC magnitude that are predictive of the epileptogenic zone. Each EEG channel constitutes a node, and its associated N-dimensional feature vector forms one row of the node feature matrix X∈R^*n*×*N*^. The graph-learning pipeline comprises three components: (i) a directed graph convolution layer based on personalised PageRank; (ii) a second-order proximity convolution capturing shared-neighbourhood structure; and (iii) a multi-scale Inception block that fuses both representations (Figure 1).

##### Graph representation and transition matrix

To construct the random-walk transition matrix, self-loops are first added to W to ensure aperiodicity, and the result is row-normalised:

(Equation 5) $$\tilde{P}={\tilde{D}}^{-1}\tilde{A},\tilde{A}=W+I$$

where $\tilde{A}=W+I$ is the adjacency matrix augmented with self-loops, $\tilde{D}$ is the corresponding diagonal degree matrix, and I is the *n*×*n* identity matrix. Each element of $\tilde{P}$ represents the transition probability from a source channel to a target channel, capturing the relative strength of directed causal influence in the weighted PDC network.

##### Personalised PageRank and stationary distribution

Because the EEG connectivity graph may contain weakly connected or isolated nodes, the random walk on $\tilde{P}$ is not guaranteed to converge to a unique stationary distribution. Following Tong et al.,^38^ an auxiliary teleport node *ξ* is introduced. Each of the *n* channel nodes transitions to *ξ* with teleport probability *α* = 0.15, and *ξ* returns uniformly to all channel nodes with probability $\frac{1}{n}$. This yields an augmented (*n*+1)×(*n*+1) transition matrix:

(Equation 6) $$P_{\text{ppr}}=\left[\begin{array}{cc} \left(1-\alpha \right)\tilde{P} & \alpha 1_{n\times 1}\\ \frac{1}{n}1_{1\times n} & 0 \end{array}\right],P_{\text{ppr}}\in R^{\left(n+1\right)\times \left(n+1\right)}$$

The construction guarantees that *P*_ppr_ is both irreducible and aperiodic, ensuring the existence of a unique left eigenvector with eigenvalue 1 (the Perron vector). The first *n* components of this eigenvector, after *ξ*_1_-normalisation, yield the approximate stationary distribution π_appr_∈R^*n*^, which encodes the long-run visit frequency of each channel node and serves as a surrogate for the degree matrix in the directed setting. The corresponding diagonal matrix Π_appr_ = diag(*π*_appr_) weights each node according to its structural importance in the directed epileptic network. The teleport probability *α* governs a theoretically grounded trade-off: smaller *α* values preserve greater directed structural information in the Laplacian, whereas larger *α* values progressively shift the operator toward an undirected symmetric form.^38^ However, empirical evaluation in the original DiGCN framework demonstrated that moderately higher *α* values improve classification accuracy, and the authors explicitly noted that *α* should be adjusted for different datasets rather than fixed at the default value of *α* = 0.1.^38^ Consistent with this dataset-dependency principle, our ablation experiments across α∈{0.05, 0.10, 0.15, 0.20, 0.25} identified *α* = 0.15 as the optimal operating point for PDC-derived epileptic brain graphs, remaining within the recommended range of α∈[0.05, 0.2] that balances directed structural retention with classification performance.

##### Directed graph convolution (first-order, k = 1)

Given $\tilde{P}$ and Π_appr_, the directed graph convolution is defined as:

(Equation 7) $$Z^{\left(1\right)}=\frac{1}{2}\left(\Pi_{\text{appr}}^{1/2}\tilde{P}\Pi_{\text{appr}}^{-1/2}+\Pi_{\text{appr}}^{-1/2}{\tilde{P}}^{T}\Pi_{\text{appr}}^{1/2}\right)X\theta^{\left(1\right)}$$

where Z^(1)^∈R^*n*×*d*^ is the convolved node representation with *d* output dimensions, and θ^(1)^∈R^*N*×*d*^ is the trainable weight matrix. The forward term $\Pi_{\text{appr}}^{1/2}\tilde{P}\Pi_{\text{appr}}^{-1/2}$ propagates features along the original directed edges, whilst the backward term $\Pi_{\text{appr}}^{-1/2}{\tilde{P}}^{T}\Pi_{\text{app}r}^{1/2}$ aggregates features from reverse-direction neighbors. Averaging the two terms symmetrises the propagation whilst retaining the directional weighting encoded in Π_appr_, thereby capturing both the sources and targets of causal influence for each channel node. This column-wise normalisation scheme—inherited from the PDC computation—makes in-degree the natural surrogate for node importance, consistent with the graph-theoretic analysis described above.

##### Second-order proximity convolution (k = 2)

Direct PDC connections capture only pairwise first-order causal relationships. To incorporate higher-order structural similarity, the second-order proximity between nodes *i* and *j* is defined via two complementary path types: a meeting path and a diffusion path, both passing through a common intermediate node *e*.^80^^,^^81^ Two channel nodes are considered second-order proximate only when both path types simultaneously exist, preventing the unbalanced receptive fields that arise from long asymmetric paths in digraphs. The maximum proximity order *k* = 2 was determined through ablation experiments, which showed no significant improvement in classification accuracy beyond second-order proximity on the present dataset, whilst higher orders introduced additional computational overhead. The second-order proximity matrix is accordingly:

(Equation 8) $$P_{ij}^{\left(2\right)}=\frac{1}{2}\left[{\left(\tilde{P}{\tilde{P}}^{T}\right)}_{ij}+{\left({\tilde{P}}^{T}\tilde{P}\right)}_{ij}\right]$$

where $P_{ij}^{\left(2\right)}$ is defined as the average of ${\left(\tilde{P}{\tilde{P}}^{T}\right)}_{ij}$ and ${\left({\tilde{P}}^{T}\tilde{P}\right)}_{ij}$ when both are strictly positive, and zero otherwise. The matrix *P*^(2)^ is symmetric by construction. After symmetric degree normalisation, the second-order convolution is:

(Equation 9) $$Z^{\left(2\right)}={\left(W^{\left(2\right)}\right)}^{-1/2}P^{\left(2\right)}{\left(W^{\left(2\right)}\right)}^{-1/2}X\theta^{\left(2\right)}$$

where $W^{\left(2\right)}=\text{diag}\left(P_{\text{row}-\text{sum}}^{\left(2\right)}\right)$ is the diagonal degree matrix of P^(2)^, and θ^(2)^∈R^*N*×*d*^ is the corresponding trainable weight matrix.

##### Multi-scale inception block and network architecture

The three feature streams are fused within a single Inception block via element-wise summation:

(Equation 10) $$Z_{I}=Z^{\left(0\right)}+Z^{\left(1\right)}+Z^{\left(2\right)},Z^{\left(0\right)}=X\theta^{\left(0\right)}$$

where *Z*^(0)^ = *X*θ^(0)^ is a linear skip connection that preserves un-smoothed node features, and θ^(0)^∈R^*N*×*d*^ is its trainable weight. The summation fusion is adopted in preference to concatenation: in deep networks, summation requires fewer parameters and thus better prevents overfitting.^38^ Three Inception blocks are stacked sequentially, with ReLU activation and dropout applied between successive blocks. This architecture allows each block to simultaneously receive information from direct PDC neighbors (first-order), shared-intermediary nodes (second-order), and the raw feature space (zeroth-order skip), yielding a receptive field that covers two-hop causal paths without requiring additional convolutional layers. The final node-embedding matrix is aggregated into a fixed-dimensional graph-level representation, which is passed to a linear classifier to predict the seizure state of the corresponding EEG epoch.

##### Model training configuration

The MVAR model order used for PDC graph construction was selected using the Akaike information criterion (AIC). The AIC-selected order of *p* = 3 was adopted as the default setting for PDC estimation. For DiGCN training, the number of graph convolutional layers was 3, the maximum proximity order was *K* = 2, the personalised PageRank teleport probability was *α* = 0.15, and dropout was set to 0.5. The classifier was trained using the Adam optimiser with a learning rate of 0.001, weight decay of 5 × 10^−4^, and a batch size of 64. Training was performed for a maximum of 100 epochs, with early stopping applied when validation accuracy did not improve for 20 consecutive epochs. The model was trained using categorical cross-entropy loss:

(Equation 11) $$L=-\frac{1}{N}\sum_{i=1}^{N}\;\log\left[\frac{\exp\left(z_{i,y_{i}}\right)}{\sum_{c=1}^{C}\;\exp\left(z_{i,c}\right)}\right]$$

where *z*_*i*,*c*_ denotes the logit output for sample *i* at class *c*, *y*_*i*_∈{0,1} is the ground-truth label (interictal = 0, preictal = 1), and *C* = 2.

#### GNNExplainer and HITS analysis

Although the DiGCN classifier provides accurate seizure-state predictions, its multi-layer message-passing architecture does not directly reveal which directed causal edges in the PDC network are responsible for each decision. To address this, we apply GNNExplainer as a post-hoc interpretability method to identify, for each preictal EEG window, the subgraph of W that most critically determines the model prediction. The resulting explanation subgraph is subsequently analyzed using the HITS algorithm to quantify the causal role of individual electrode channels within the identified epileptic connectivity pattern (Figure 1).

##### Edge mask optimisation

Given the trained and frozen DiGCN classifier Φ and a preictal graph instance with adjacency matrix W and node feature matrix X, GNNExplainer learns a real-valued edge mask M∈R^*n*×*n*^. The mask is passed through a sigmoid activation to produce a soft selection *σ*(M)∈[0, 1]^*n*×*n*^, which is applied element-wise to the original weighted adjacency matrix to obtain the masked graph:

(Equation 12) $${\tilde{A}}_{S}=W⊙\sigma \left(M\right),M\in R^{n\times n}$$

where ⊙ denotes element-wise multiplication. Unlike the original GNNExplainer formulation in which the mask directly replaces the adjacency matrix, our construction multiplies the sigmoid mask by W so that the continuous PDC weights are preserved in the masked graph. This ensures that ${\tilde{A}}_{S}$ retains the graded causal strength of each selected edge, rather than treating all retained edges as equally important.

##### Objective function

The mask M is optimised by minimising a composite loss function

(Equation 13) $$L=L_{\text{pred}}+\lambda_{1}L_{\text{size}}+\lambda_{2}L_{\text{ent}}$$

The three terms are defined as follows. The prediction loss *L*_pred_ maximises the model’s confidence in the predicted class ŷ under the masked graph:

(Equation 14) $$L_{\text{pred}}=-\log P_{\Phi }\left(\hat{y}|{\tilde{A}}_{S},X\right)$$

The size regularisation term *L*_size_ penalises the total mass of the mask, encouraging a sparse explanation that retains only the most influential edges:

(Equation 15) $$L_{\text{size}}=\frac{1}{n^{2}}\sum_{i,j}\;\sigma \left(M_{ij}\right)$$

The entropy regularisation term *L*_ent_ promotes discreteness of the mask entries by penalising values that remain near 0.5, pushing each edge selection toward a hard include-or-exclude decision:

(Equation 16) $$L_{\text{ent}}=\frac{1}{n^{2}}\sum_{i,j}\;\left[-\sigma \left(M_{ij}\right)\log\sigma \left(M_{ij}\right)-\left(1-\sigma \left(M_{ij}\right)\right)\log\left(1-\sigma \left(M_{ij}\right)\right)\right]$$

where *M*_*ij*_ is the (*i*, *j*)-th entry of M. The mask is optimised via Adam gradient descent with an early-stopping criterion based on the validation loss plateau.

##### Edge importance extraction

Upon convergence, the optimal mask *σ*∗(M) is combined with the original PDC weights to produce the edge importance matrix:

(Equation 17) $$e_{ij}=\sigma^{*}\left(M_{ij}\right)\cdot w_{ij}$$

Each element *e*_*ij*_ jointly reflects the structural importance assigned by the explainer and the underlying causal influence strength encoded in *w*_*ij*_. Entries with *e*_*ij*_ = 0 correspond to edges that either received negligible mask weight or were absent in the original PDC graph. To further concentrate the explanation on the most salient directed edges, a percentile-based threshold is applied to the non-zero entries, retaining only edges whose importance value exceeds the threshold, generating the final explanation adjacency matrix *A*_expl_. The retention proportion was determined by evaluating directional concordance of eight graph-theoretic topology metrics between explanation subgraphs and the original PDC networks across retention proportions of 10%–50%. Three metrics—betweenness centrality, reciprocity, and degree distribution variance—exhibited direction reversals below 30%: excessive sparsification collapses multi-hop EZ relay paths (deflating betweenness), preferentially retains the high-weight bidirectional edges that GNNExplainer scores highest (spuriously elevating reciprocity), and reduces most nodes to near-zero degree irrespective of neurophysiological identity (collapsing the hub-periphery contrast that drives variance elevation). In each case the reversal is a structural consequence of graph sparsification rather than a neurophysiological signal. The 30% threshold was therefore adopted as the minimum proportion at which all eight metrics maintained directional concordance with the full PDC network simultaneously, maximising explanation sparsity subject to the constraint of topological fidelity.

##### Hub–authority analysis on the explanation subgraph

The explanation adjacency matrix *A*_expl_ defines a weighted directed subgraph over the *n* electrode channels. To quantify the functional role of each channel within this subgraph, the HITS (Hyperlink-Induced Topic Search) algorithm^82^ is applied iteratively:

(Equation 18) $$h^{\left(t+1\right)}=A_{\text{expl}}a^{\left(t\right)},a^{\left(t+1\right)}=A_{\text{expl}}^{T}h^{\left(t\right)}$$

where h^(t)^∈R^*n*^ and a^(t)^∈R^*n*^ are the hub and authority score vectors at iteration *t*, respectively. After each iteration, both vectors are L_2_-normalised. The algorithm is run for a maximum of 100 iterations or until convergence, defined as a change in both vectors below 10^−6^. A high *hub score* for channel *i* indicates that *i* transmits strong causal influence to many high-authority channels, characterising it as a key driver of network dynamics. A high *authority score* for channel *i* indicates that *i* receives strong directed input from many high-hub channels, identifying it as a convergent target of causal inflow—a property consistent with the role of epileptogenic nodes that accumulate pathological driving signals from the surrounding network prior to seizure onset.

### Quantification and statistical analysis

Statistical analyses were performed on 8,160 observation-level epochs nested within 68 patients from the held-out test set (4,080 preictal and 4,080 interictal). To account for the within-patient dependence among repeated epoch-level measurements, a linear mixed-effects model was fitted with patient identity as a random intercept. Statistical analyses were restricted to the held-out test set to minimise the potential influence of model training on the inference procedure. To assess the statistical significance of performance differences across all pairwise baseline comparisons, McNemar’s test was applied across all three datasets, with Holm–Bonferroni correction to control the family-wise error rate.
