# Supplementary material for: Directed graph neural networks with partial directed coherence for seizure prediction and epileptogenic network characterization
Source: iScience. 2026 Aug 10;29(8):117068. doi: 10.1016/j.isci.2026.117068 (PMC13486768; doi:10.1016/j.isci.2026.117068)
Supplement: Supplementary file 1 [file mmc1.pdf]

iScience, Volume *29*

Supplemental information

Directed graph neural networks with partial directed coherence for seizure prediction and epileptogenic network characterization

Chenshuo Zhao, Hui Huang, Chao Fang, Hong Zhang, and Suyue Zheng

Table S1. Reproducibility analysis of PDC-DiGCN across ten independent random seed configurations. Per-seed results are reported as mean ± SD (%) over five runs.

Seed	Accuracy	Precision	Recall	F1-Score
42	97.28 ± 0.30	96.22 ± 0.34	98.30 ± 0.32	97.25 ± 0.31
123	97.66 ± 0.46	97.83 ± 0.47	97.37 ± 0.44	97.60 ± 0.48
456	98.34 ± 0.50	99.06 ± 0.50	97.53 ± 0.52	98.29 ± 0.51
789	97.96 ± 0.46	97.55 ± 0.39	98.30 ± 0.41	97.92 ± 0.40
1234	97.74 ± 0.40	97.83 ± 0.35	97.53 ± 0.44	97.68 ± 0.42
2024	98.19 ± 0.44	97.70 ± 0.46	98.61 ± 0.43	98.15 ± 0.46
2025	97.74 ± 0.33	98.28 ± 0.35	97.06 ± 0.29	97.67 ± 0.32
3407	96.91 ± 0.31	98.87 ± 0.30	94.75 ± 0.35	96.76 ± 0.34
5678	97.43 ± 0.54	96.79 ± 0.61	97.99 ± 0.50	97.39 ± 0.58
9527	98.04 ± 0.34	99.21 ± 0.30	96.75 ± 0.33	97.97 ± 0.32
Average	97.73 ± 0.43	97.93 ± 0.96	97.42 ± 1.11	97.67 ± 0.45

Table S2. Per-patient seizure prediction performance on the CHB-MIT Scalp EEG Database. Data are reported as mean ± SD (%) over five runs.

Patient ID	Accuracy (%)	Sensitivity (%)	Specificity (%)	F1-Score (%)
chb01	95.94 ± 0.80	92.61 ± 2.01	99.12 ± 0.45	95.69 ± 0.89
chb02	96.48 ± 0.54	94.56 ± 1.62	98.32 ± 0.54	96.32 ± 0.61
chb03	96.00 ± 0.51	92.95 ± 1.53	98.91 ± 0.55	95.77 ± 0.58
chb04	96.50 ± 0.44	94.81 ± 1.18	98.11 ± 0.43	96.35 ± 0.48
chb05	95.85 ± 0.84	92.18 ± 1.75	99.54 ± 0.25	95.70 ± 0.90
chb06	96.02 ± 0.72	93.29 ± 2.38	98.61 ± 0.92	95.80 ± 0.83
chb07	97.11 ± 0.90	95.10 ± 2.63	99.15 ± 1.18	97.05 ± 0.96
chb08	96.05 ± 0.96	94.68 ± 3.03	97.35 ± 1.61	95.88 ± 1.08
chb09	95.58 ± 0.85	92.06 ± 2.18	98.94 ± 0.45	95.30 ± 0.96
chb10	96.18 ± 0.73	93.23 ± 2.11	99.00 ± 0.71	95.96 ± 0.82
chb11	95.89 ± 0.59	93.45 ± 1.99	98.23 ± 1.29	95.69 ± 0.67
chb12	97.39 ± 0.55	95.77 ± 1.44	99.03 ± 0.44	97.36 ± 0.58
chb13	96.21 ± 0.49	93.32 ± 1.29	99.12 ± 0.34	96.11 ± 0.53
chb14	94.72 ± 1.11	91.38 ± 3.50	97.91 ± 1.21	94.38 ± 1.31
chb15	97.15 ± 0.49	95.05 ± 1.44	99.28 ± 0.52	97.10 ± 0.52
chb16	95.73 ± 0.65	92.24 ± 1.75	99.06 ± 0.41	95.46 ± 0.73
chb17	95.77 ± 0.44	93.69 ± 2.09	97.76 ± 1.31	95.58 ± 0.52
chb18	94.37 ± 1.01	89.58 ± 2.93	98.94 ± 0.82	93.93 ± 1.18
chb19	95.41 ± 0.88	92.06 ± 3.25	98.61 ± 1.53	95.12 ± 1.03
chb20	95.55 ± 1.00	92.98 ± 2.83	97.99 ± 1.08	95.31 ± 1.15
chb21	97.27 ± 0.52	95.39 ± 1.66	99.18 ± 0.67	97.23 ± 0.55
chb22	96.26 ± 0.89	93.94 ± 1.71	98.47 ± 0.20	96.07 ± 0.98
chb23	95.92 ± 0.82	92.90 ± 2.00	98.97 ± 0.71	95.80 ± 0.89
chb24	97.31 ± 0.77	95.46 ± 2.01	99.18 ± 0.54	97.26 ± 0.83
Average	96.11 ± 0.76	93.45 ± 1.49	98.70 ± 0.56	95.93 ± 0.86

Table S3. Per-patient seizure prediction performance on the Siena Scalp EEG Database. Data are reported as mean ± SD (%) over five runs.

**Patient ID**	**Accuracy (%)**	**Sensitivity (%)**	**Specificity (%)**	**F1-Score (%)**
PN00	95.44 ± 0.38	92.49 ± 0.96	98.26 ± 0.49	95.19 ± 0.43
PN01	96.95 ± 0.27	95.67 ± 0.31	98.17 ± 0.65	96.84 ± 0.27
PN03	97.07 ± 1.60	94.71 ± 3.52	99.46 ± 0.43	96.98 ± 1.72
PN05	95.83 ± 1.35	92.33 ± 3.26	99.36 ± 0.61	95.67 ± 1.45
PN06	95.73 ± 0.81	92.33 ± 1.70	99.15 ± 0.31	95.59 ± 0.87
PN07	97.20 ± 0.93	94.84 ± 2.18	99.58 ± 0.39	97.13 ± 0.98
PN09	96.95 ± 0.44	94.83 ± 0.80	99.09 ± 0.14	96.89 ± 0.46
PN10	95.70 ± 1.65	92.80 ± 4.18	98.47 ± 1.14	95.42 ± 1.91
PN11	95.85 ± 0.76	92.27 ± 1.51	99.45 ± 0.18	95.70 ± 0.82
PN12	96.13 ± 0.48	94.17 ± 1.98	98.12 ± 1.64	96.07 ± 0.52
PN13	97.08 ± 0.91	94.54 ± 1.93	99.65 ± 0.19	97.01 ± 0.98
PN14	96.25 ± 0.69	93.59 ± 1.45	98.94 ± 0.25	96.16 ± 0.74
PN16	95.26 ± 0.96	91.16 ± 2.13	99.39 ± 0.30	95.06 ± 1.06
PN17	97.03 ± 0.65	94.44 ± 1.39	99.67 ± 0.17	96.97 ± 0.69
Average	96.32 ± 0.70	93.58 ± 1.34	99.05 ± 0.57	96.19 ± 0.76
